# Antioxidant Properties of Plant-Derived Phenolic Compounds and Their Effect on Skin Fibroblast Cells

**DOI:** 10.3390/antiox10050726

**Published:** 2021-05-05

**Authors:** Anna Merecz-Sadowska, Przemysław Sitarek, Ewa Kucharska, Tomasz Kowalczyk, Karolina Zajdel, Tomasz Cegliński, Radosław Zajdel

**Affiliations:** 1 Department of Computer Science in Economics, University of Lodz, 90-214 Lodz, Poland; radoslaw.zajdel@uni.lodz.pl; 2 Department of Biology and Pharmaceutical Botany, Medical University of Lodz, 90-151 Lodz, Poland; przemyslaw.sitarek@umed.lodz.pl; 3 Chair of Gerontology, Geriatrics and Social Work at the Faculty of Pedagogy, Ignatianum Academy in Cracow, 31-501 Cracow, Poland; ewa.kucharska@vadimed.com.pl; 4 Department of Molecular Biotechnology and Genetics, University of Lodz, 90-237 Lodz, Poland; tomasz.kowalczyk@biol.uni.lodz.pl; 5 Department of Medical Informatics and Statistics, Medical University of Lodz, 90-645 Lodz, Poland; karolina.smigiel@umed.lodz.pl (K.Z.); tomasz.ceglinski@umed.lodz.pl (T.C.)

**Keywords:** plants, phenolic compounds, antioxidant properties, ROS, fibroblasts

## Abstract

Plants are rich sources of a diverse range of chemicals, many of which have significant metabolic activity. One large group of secondary compounds are the phenolics, which act as inter alia potent reactive oxygen scavengers in cells, including fibroblasts. These common dermis residue cells play a crucial role in the production of extracellular matrix components, such as collagen, and maintaining the integrity of connective tissue. Chronic wounds or skin exposure to UV-irradiation disrupt fibroblast function by the generation of reactive oxygen species, which may damage cell components and modify various signaling pathways. The resulting imbalance may be reversed by the antioxidant activity of plant-derived phenolic compounds. This paper reviews the current state of knowledge on the impact of phenolics on fibroblast functionality under oxidative stress conditions. It examines a range of compounds in extracts from various species, as well as single specific plant-derived compounds. Phenolics are a good candidate for eliminating the causes of skin damage including wounds and aging and acting as skin care agents.

## 1. Introduction

Plants are a rich source of structurally diverse chemical compounds. Nowadays, these chemical compounds are of great interest in several fields of science including pharmacy and cosmetology. Several therapeutic benefits have been attributed to naturally derived materials based on plant secondary compounds. The first written report of the usage of plants for therapeutic purposes dates back 5000 years [1]. Herbal medicine has been applied for various ailments, and at least 31 plants are known to be effective in treating skin problems ranging from rashes to cancer [2]. Interestingly, cosmetology has also recently seen a tendency to return to herbal formulas [3].

Special attention has been paid to the effectiveness of scavengers of reactive oxygen species (ROS), and studies have found phenolic compounds, the most widespread class of plant secondary metabolites, to be particularly good scavengers of ROS [4]. Phenolic compounds are able to modulate numerous signaling pathways related to cell division, cycle arrest, autophagy, apoptosis, and inflammation on numerous cell lines, including non-melanoma and melanoma skin cancer [5]. Despite this, further research into the quality, safety, molecular mechanism of action, and clinical efficacy of numerous plant-derived chemical compounds is still needed. Only a few studies have been performed on normal cells, such as fibroblasts; however, their findings indicate they have enormous potential in supporting the functions of the most important dermis resident cells.

The skin, the largest organ of the human body, is divided into three layers: epidermis, dermis, and subcutaneous tissue. The middle one, the dermis, is mostly composed of fibroblasts. Fibroblasts are a heterogenous group of cells, responsible for the production of extracellular matrix (ECM) components, particularly collagen. Their primary function is to create a structural scaffold for tissues and organs, and to maintain integrity within the connective tissue. Therefore, they are crucial in the wound healing process. Following injury, under physiological conditions, fibroblasts activate and differentiate into myofibroblasts to allow greater production of ECM components and begin wound closure [6]. They are also responsible for the release of elastic fibers, which prevent the signs of photoaging [7].

However, their functions may be disrupted by a loss of cellular redox homeostasis due to by elevated levels of ROS and oxidative stress. Normally, ROS are generated by mitochondrial respiration [8], but under pathological conditions, ROS production is increased by chronic wounds and production by phagocytes [9], as well as by exposure to UV radiation, via various mechanisms [10]. ROS broadly affect cellular components, and damage proteins, DNA and lipids, and the cellular response can be altered by cross talk between ROS and various signaling pathways [11]. This reduction of fibroblast functionality results in disturbed wound healing and the emergence of clinical aging features.

Fibroblast function can be restored with treatment with antioxidants, such as plant-derived phenolic compounds. Fibroblasts exposed to phenolics, in particular, decrease ROS production [12] and increase collagen expression [13]. In vitro studies also indicate that these chemicals are able to modify several biomolecular pathways in cells [5]. They have been found to mediate protection against oxidants via stimulation of nuclear factors (erythroid-derived 2), factor 2 (Nrf2) [14] and inhibition of mitogen-activated protein kinases (MAPKs); they suppress the expression of matrix metalloproteinases (MMPs), which are known to disrupt collagen and limit nuclear factor kappa-light-chain-enhancer of activated B cells (NF-kB) signaling and pro-inflammatory agent release [15]. These secondary metabolites are a promising tool for skin care.

This paper reviews the properties of selected plant extracts from various species rich in phenolic compounds, as well as isolated phenolics, on fibroblast cells. It examines the potential of phenolics as ROS scavengers and as important agents for mediating wound healing and anti-aging processes under oxidative stress conditions.

## 2. Criteria for the Selection of Experimental Papers

This paper reviews works published between 2010 and 2020 aimed at assessing the impact of phenolic compounds on dermal fibroblasts. The studies were selected from the electronic databases PubMed/MEDLINE, Scopus, Web of Science, and Google Scholar. The search terms included phenolic compounds, plant extracts, plant-derived phenolic compounds, and fibroblasts. Published experimental studies of plant extracts with phenolic content or phenolic-derived compounds which demonstrate in vitro activity on dermal fibroblast cells were analyzed. Data on the in vivo effects in animal or human models were also included, but only if the paper included an evaluation of the in vitro efficacy of the phenolic extracts or compounds on fibroblasts.

The following articles were excluded: papers reporting review articles, those published in languages other than English, those with only an abstract or lacking full text access, those lacking specific plant names with no report of clear objectives and methodologies, those published before 2010, those using cell lines other than fibroblasts, or those based on plant-derived compounds other than phenolics. Any duplicates of articles obtained from the electronic databases were removed. After removal, the inclusion and exclusion criteria were checked. Each selected document was examined.

The following data regarding the plant extracts with phenolic content was collected in a table: the name of the family, the scientific names of the species, parts of the plants used for extract preparation, types of extract, compounds identified in the extracts, fibroblast cell line and the mechanisms of action, as well as the reference to the paper. Articles describing isolated plant phenolic compounds were discussed in the main text. The names of the plants have been verified according to most recent taxonomy through https://mpns.science.kew.org/mpns-portal/searchName (accessed on 15 March 2021).

## 3. Characteristics and Function of Phenolic Compounds—Crucial Plant Secondary Metabolites with Antioxidative Properties

The plant kingdom is a rich source of secondary metabolites that protect against biotic and abiotic threats, such as pathogens and radiation, floods, heavy metals or changes in climate [16,17]. Several classes of plant secondary metabolites are known, including phenolics, alkaloids, saponins, terpenes, lipids, and carbohydrates. These have a wide range of biological effects in humans, including antioxidant [18], anti-inflammatory [19], anticancer [20], antibacterial [21], antiviral, antifungal [22], antiobesity [23], antidiabetic, antiosteoporotic, and cardioprotective or neuroprotective [24] properties.

Phenolic compounds are those characterized by the presence of at least one phenol group; however, they are known to demonstrate about 8000 different skeleton structures. The group can be subdivided into simple phenolics, tannins, coumarins, flavonoids, chromones and xanthones, stilbenes, and lignans based on their structure. Simple phenolics contain a single C6 phenyl ring with one or more hydroxyl, aldehydic, or carboxylic groups attached. Tannins are composed of phenyl rings with two or three hydroxyl groups, which can complex with proteins and carbohydrates. Coumarins are derivatives of benzo-α-pyrones (lactones) composed of C6-C3 carbon skeleton and exist in free form or condensed with sugars as glycosides [25].

The most numerous subgroup is the flavonoids, consisting of more than 6000 different compounds. They possess C6-C3-C6 basic carbon skeleton organized as closed pyran ring C as central carbon chain and two benzene rings A and B are linked in position 2, 3, or 4 of the C ring. Isoflavones and neoflavonoids are a classes of flavonoids in which the B ring is attached in 3 and 4 position of the C ring, respectively. Those in which the B ring is linked in position 2 may be further subdivided into several classes, including flavones, flavonols, flavanones, flavanonols, flavanols or catechins, anthocyanins, and chalcones, depending on the basis of the structural features of the C ring [26].

Two other commonly appearing groups are the chromones and xanthones, which are derivatives of benzo-γ-pyrone. Naturally occurring chromones are composed of a C6-C3 basic carbon skeleton with methyl or alkyl substituents at position 2 of the ring and hydroxyl or alkoxyl substituents at positions 5 and 7, whereas xanthones are composed of a C6-C1-C6 carbon structure with various substituents. In addition, stilbenes (C6-C2-C6) are found mostly in the heartwood of various plant species and lignans (C6-C3), formed by dimerization of two phenylpropene derivatives, are also commonly found [27,28,29].

Many of these compounds possess free radical scavenging properties deriving from the presence of hydroxyl (-OH) and methoxy (-OCH3) groups in their molecules [30]. Numerous plant families are known as a rich source of phenolic compounds, including the Asteraceae, Rosaceae, and Lamiaceae [31].

A considerable body of evidence indicates that phenolic compounds exert spectacular antioxidant effects on human fibroblast cells. They are found to elevate fibroblasts viability and motility [32,33]. Moreover, plant-derived phenolics may have a protective effect on skin exposed to UV radiation [34] and air pollution [35].

## 4. Fibroblasts as Important Dermis Resident Cells and Their Characterization

**Figure 1:**
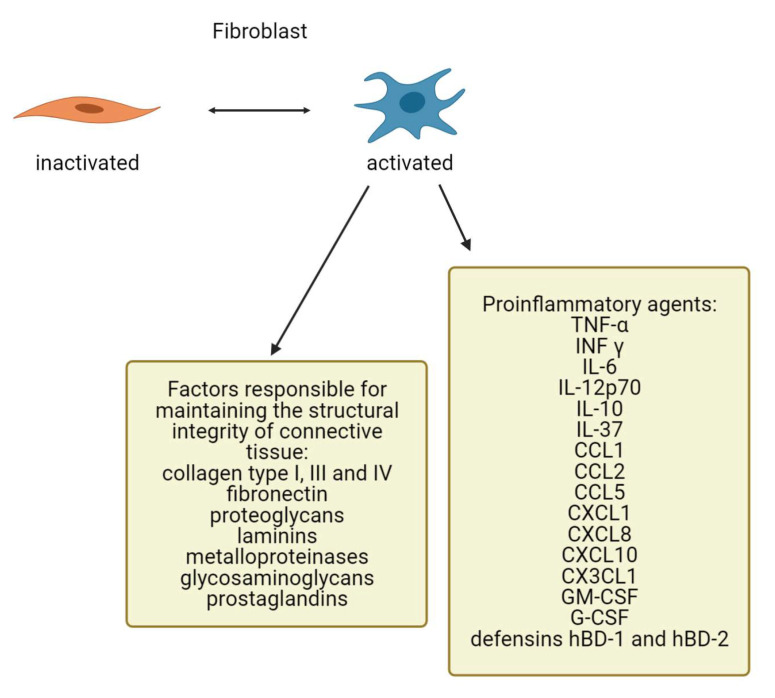
Expression of ECM components and proinflammatory factors by fibroblasts (created by BioRender.com).

Fibroblasts are common stromal cells present in human connective tissue. They have a heterogenous morphology characterized by an elongated spindle shape or stellate shape specific for inactive and active cells, respectively. Fibroblasts, and other mesenchymal cells, are believed to be derived by epithelial–mesenchymal transition (EMT), i.e., the loss of epithelial cells followed by reduced expression of their specific molecular markers such as cytokeratins or E-cadherin, and elevated expression of certain proteins by fibroblasts. Conversely, in some situations like wound healing and tumorigenesis, fibroblasts may give rise to epithelial cells via mesenchymal to epithelial transition (MET). Fibroblasts have been isolated from heart, lung, gastrointestinal tract, muscles, and the dermis. They produce several factors responsible for maintaining the structural integrity of the connective tissue, including collagen type I, III, and IV, fibronectin, proteoglycans, laminins, metalloproteinases, glycosaminoglycans, and prostaglandins (Figure 1). Due to their diversity, fibroblasts perform a variety of functions in different organ systems and in various dermal locations [36,37,38,39].

The skin, the largest organ of the body, is divided into an epidermis and a dermis. The underlying dermis, composed of papillary and reticular layers, consists primarily of fibroblasts. The progenitors of upper dermis fibroblasts give rise to papillary fibroblasts and hair follicle dermal papillae, which control hair growth, and arrector pili muscles, which regulate piloerection. Papillary fibroblasts are characterized by higher proliferation rates and a lack of adipogenic potential. The progenitors of the lower dermis fibroblasts give rise to reticular fibroblasts and adipocytes belonging to the hypodermis. In contrast, the reticular cells demonstrate lineage-less proliferative potential, and the ability to undergo adipogenesis [40,41]. Different dermal fibroblast subpopulations vary with regard to gene expression. Papillary variants express platelet derived growth factor receptor (PDGFR-α)- alpha and dipeptidyl peptidase-4 (DPP4) as markers, whereas reticular cells express PDGFR-α, delta-like noncanonical Notch ligand 1(DLK1) and spinocerebellar ataxia type 1 (SCA1) [42].

The primary function of dermal fibroblasts is to produce important compounds for ECM formation. The ECM can provide an environment for many cell types and confers structural support. It is composed of fiber-forming molecules including collagen, fibrin, fibronectin, elastin, vitronectin, and fibrillin, as well as nonfiber-forming molecules, particularly proteoglycans and glycosaminoglycans [43]. The most common fiber-forming protein is collagen, which comprises 77% of the fat-free dry weight of human skin [44]. Fibroblasts express different ratios of procollagen type I and III mRNA depending on their depth in the dermis; in addition, those located deeper produce less collagenase mRNA than those located in more superficial layers [45].

The ECM also plays a role in the regulation of healing. The production of new ECM is crucial for wound closure by acting as a scaffold and enabling the migrated cells to participate [46,47]. The functionality of the ECM is threated by age-related changes. Young skin fibroblasts adhere to the dermal ECM and secrete primarily type I collagen. However, the fragmentation of collagen associated with aging results in lowered adherence and general weakening. Aged fibroblasts produce more MMPs and less ECM components, resulting in additional collagen fibril fragmentation and the potential disruption of skin homeostasis [48,49].

Fibroblasts may also participate in skin immunity. They express the toll-like receptors (TLRs) TLR-1 to TLR-10 and can sense microorganisms or their components. As a consequence, the cells can release a number of proinflammatory cytokines upon stimulation: chemokines, growth factors, or antimicrobial peptides like tumor necrosis factor (TNF)-α, interferon (INF) γ, interleukin (IL) -6, IL-12p70, and IL-10, C-C motif chemokine ligand 1 (CCL1), C-C motif chemokine ligand 2 (CCL2), C-C motif chemokine ligand 5 (CCL5), C-X-C motif chemokine ligand 1 (CXCL1), C-X-C motif chemokine ligand 8 (CXCL8), C-X-C motif chemokine ligand 10 (CXCL10), and C-X3-C motif chemokine ligand 1 (CX3CL1), granulocyte/macrophage colony-stimulating factor (GM-CSF) and granulocyte colony-stimulating factor (G-CSF), IL-37, defensins hBD-1, and hBD-2 [50] (Figure 1). Additionally, cytokines such as IL-1, IL-4, IL-10, IL-13, TNFα, and transforming growth factor (TGF)-β have been identified to influence fibroblast activation [51].

Additionally, fibroblasts express the chemokine C-X-C motif chemokine ligand 12 (CXCL12), which binds to the C-X-C motif chemokine receptor 4 (CXCR4) and promotes Langerhans cell migration to the dermis [52]. In response to TLR signaling, fibroblasts also produce serum amyloid A, which is able to induce numerous proinflammatory agents from various immune cells [53]. Hyperthermic stress has also been found to stimulate the release of TNFα, IL-1β, IL-6, IL-8, and IL-25 from human fibroblasts in vitro [54].

## 5. The Impact of Reactive Oxygen Species on Dermal Fibroblasts

ROS, highly reactive molecules with unpaired electrons, play an important role in skin cells. ROS are primarily produced in human skin by oxidative phosphorylation in the respiratory chain in the mitochondria, mostly by NADPH oxidase activity. Although physiological levels of ROS are necessary for the maintenance of skin function, excessive levels are harmful [55]. In fibroblasts, excessive ROS levels result in inactivation of antioxidants including catalase (CAT), superoxide dismutase (SOD), or glutathione peroxidase (GPx). Excessive production or impaired neutralization can induce oxidative stress, resulting in direct cell damage or impaired response via crosstalk with signaling pathways [56]. In fibroblasts, increased ROS release can disrupt collagen biosynthesis and activate collagenase [57] leading to the elastin deposition characteristic of photoaged skin [58]. Additionally, ROS overproduction increases MMPs levels and decreases tissue inhibitors of metalloproteinase (TIMPs) levels [59]. Oxidative stress impairs fibroblast functionality and is linked to impaired tissue repair and aging.

High levels of ROS depend on cell cycle position induced fibroblast apoptosis, or senescence, a fundamental stress response reaction. Apoptosis is more common during the S-phase, and senescence during G1 or G2/M. Senescence is a state of irreversible cell cycle arrest. Programmed cell death is characterized by a higher level of p53, an absence of p21, in contrast to the cellular senescence state [60]. Senescent cells accumulate exponentially, contributing to tissue dysfunction. They are characterized by a special secretory phenotype that release proinflammatory factors, ECM remodeling proteases, and growth factors that exert a negative impact on the surrounding area [61]. Transiently induced senescence is required for acute wound repair, while chronic senescence is widely implicated in tissue pathology. Sustained senescence is believed to contribute to wound chronicity due to continuing inflammation [62] and impaired diabetic healing [63]. Senescence is strictly related to cell age. Aging significantly reduces the capacity of human dermal fibroblasts (HDFs) to respond to ROS. Specifically, HDFs demonstrate decreased cell viability and greater entrance into a senescent state compared with their younger counterparts [64,65].

Several ROS detoxifying enzymes as well as antioxidant proteins are induced by Nrf2. Nrf2 belongs to the basic leucine zipper transcription factor family. The agonist of Nrf2 is named Kelch-like ECH associated protein 1 (Keap1). Binding Keap1 to Nrf2 results in their degradation by proteasomes. The presence of ROS prevents the formation of Nrf2-Keap1 and enables the translocation of Nrf2 into the nucleus. The dimerization of Nrf2 and small musculoaponeurotic fibrosarcoma (MAF) proteins results in binding to DNA regions called antioxidant response elements (AREs), and transcription of the targeted genes [66]. Nrf2-deficient dermal fibroblasts are characterized by slight ROS overproduction and may lead to senescence [67]. Studies with irradiated Nrf2-deficient immortalized mouse embryonic fibroblast cells revealed an elevated basal level of ROS, which increased significantly five days after exposure [68].

**Figure 2:**
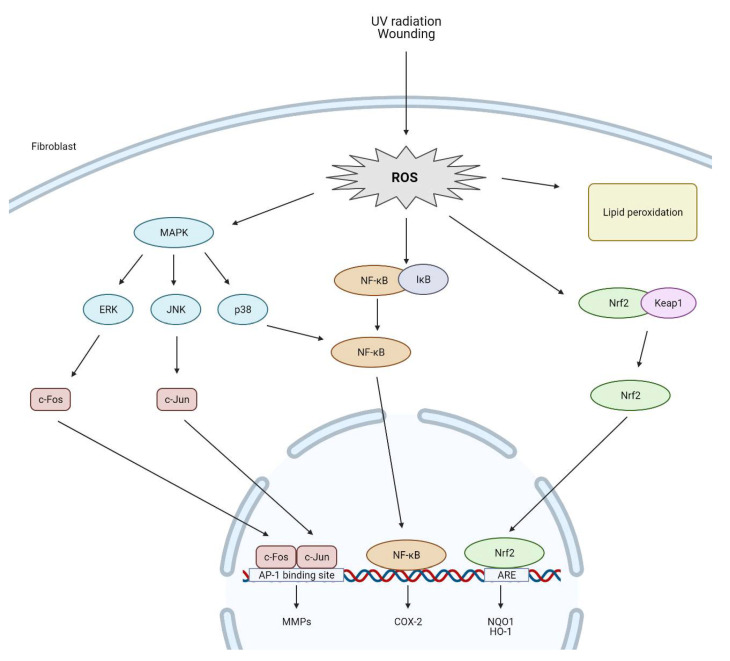
ROS-mediated activation of cell signaling pathways in dermal fibroblasts (created by BioRender.com).

Additionally, excessive ROS levels increase MMPs and decrease TIMPs [59,69]. MMPs are an important class of enzymes that promote the degradation of collagen and other proteins in the ECM [70]. Up-regulation of MMPs expression and collagen degradation is linked to the activation of the MAPK signaling pathway under oxidative stress conditions. JNK, p38, and ERK1/2 are types of MAPKs and belong to the serine/threonine kinase family involved in the transmission of external stimuli into the cell nucleus [71,72]. ERK and JNK recruit c-Fos and c-Jun to the nucleus, followed by AP-1 transcription factor activation; however, activation of p38 and inhibitory kappa kinase (IKK) is crucial for the transcriptional induction of NF-kB. Both AP-1 and NF-kB are important in regulating numerous genes that take part in in cell cycle regulation, cell proliferation, and apoptosis and the pathogenesis of inflammation [73]. Fibroblasts cultured in MMP-1 displayed reduced cell size as well as increased ROS production in comparison to those cultured in collagen gels [74]. Catalase restores the expression of collagen and TIMP-1 as well as abrogates increased expression of MMP-1, MMP-2 in ROS treatment fibroblast cells [57]. The impact of ROS on fibroblast cells are presented in Figure 2.

Aerobic organisms are equipped with enzymatic and nonenzymatic antioxidants that neutralize ROS. To elevate the effectiveness of nonenzymatic one, increasing attention has been focused on the development of novel antioxidants with natural origins. Phenolic compounds have a potent radical-scavenging properties and treatment significantly supports the function of fibroblasts. Additionally, their application to the skin surface protects fibroblasts from ROS-mediated damage. Many such phenolic compounds may be used as skin care agents [75].

Table 1 presents several plant extracts, particularly phenolics, and their in vitro effect on dermal fibroblast cells and ROS production.

In studies presented in Table 1, quantification of intracellular ROS were evaluated in the L929, 3T3 and HDFs fibroblasts. Incubation with extracts derived from *Annona muricata*, *Fuchsia magellanica*, *Pourthiaea villosa*, and *Populus nigra* did not cause cell cytotoxicity but reduced ROS release from fibroblasts. The test for ROS release was performed using dichlorodihydrofuorescein diacetate (DCFH-DA) staining. Cells were pre-treated with the extracts and then exposed to the ROS-inducing stressor (H2O2 or AAPH). The ROS detection reagent, DCFH-DA, was added and detection for ROS was performed by fluorimetry [76,77,79,80]. All extracts inhibited intracellular ROS production by protecting against oxidative stress. Antioxidant activity of the *Oryza sativa* extract was examined by ferric reducing ability of plasma (FRAP) test and confirmed their crucial free radicals scavenging properties [78]. Additionally, the transcriptional effect of *Populus nigra* extract on HDFs indicates that extract modulated the expression of CAT antioxidant enzyme gene [80]. Moreover, the expression of SOD antioxidant enzyme gene and MMP-1, MMP-3, MMP-9 collagen degradation genes in HDFs were induced and inhibited by the incubation with *Pourthiaea villosa* extract, respectively.

In two of the articles presented above, the effectiveness of plant extracts in a human/animal model was also assessed. That first study included human volunteers that applied rice panicle extract to the inner forearm twice daily for 84 days. Significantly increased skin hydration was observed followed by smoothing and anti-wrinkle effects [78]. Another study examined Swiss mice treated with *Annona muricata* extract by topical application to the inner and outer surfaces of the right ear, with a polypropylene tip. Extract treatment significantly reduced ear edema as well as histological parameters of inflammation after six hours [76].

## 6. Modulation of ROS Levels in Fibroblasts by Phenolic Compounds and Their Role in Regulation of the Wound Healing Process

Cutaneous wound healing is a physiological process that provides tissue regeneration by collaboration of several cell types. The healing process is divided into three overlapping phases: inflammatory, proliferative, and remodeling. The inflammatory response is the first consequence of tissue injury. Inflammation is essential for tissue repair. In the inflammatory phase, activation of proinflammatory cytokines and chemokines at the site of injury leads to the recruitment of immune cells such as leukocytes, as well as subtypes like neutrophils and monocytes. Infiltrating cells release another portion of cytokines and growth factors that stimulate regeneration. Monocytes convert into macrophages that remove death cells and or cell debris. Inflammatory cells release ROS that facilitate clean-up [81].

During the proliferative stage, endothelial cells, macrophages, and fibroblasts close the wound and form granulation tissue and blood vessels. Groups of fibroblasts in the granulation tissue, mainly stimulated by TGF-β, may differentiate into myofibroblasts that express smooth muscle actin. Myofibroblasts demonstrate more efficient ECM production. The subpopulation can then undergo apoptosis and is replaced by fibroblasts that promote tissue remodeling [82].

The remodeling phase is characteristic of ECM reorganization, transformation of type I to type III collagen, and its maturation by increasing the number of cross-links between fibers. Scar tissue is formed. After restoration of tissue architecture, the inflammation should be resolved, and inflammatory cells disappear. However, chronic inflammation fails to progress to healing: excess infiltration of leukocytes and macrophages, generation of proinflammatory cytokines and ROS result in the degradation of growth factors and ECM proteins essential for regeneration [83,84].

During normal wound healing, ROS act as pivotal secondary messengers. They possess the ability to enhance fibroblast proliferation and migration and promote the expression of fibroblast growth factor (FGF) and collagen production, thus facilitating ECM production and wound closure. The study revealed that the level of mitochondrial ROS in fibroblasts may influence the expression of the genes taking part in wound healing. The following genes are upregulated: those encoding collagen type III alpha 1 chain; collagen type V alpha 2 chain; GM-CSF, controlling granulocytes and macrophage functions; cathepsin K, involved in bone remodeling and resorption; insulin like growth factor 1 (IGF1), mediating growth and development; integrin subunit alpha 1 (ITGA1), one of the subunits for laminin and collagen receptor; matrix metallopeptidase 9 (MMP-9), engaged in breakdown of ECM. The following genes are blocked: actin alpha 2 (ACTA2), one of the actin proteins that participate in cell motility and intercellular signaling; angiopoietin 1, involved in vascular development and angiogenesis; monocyte chemotactic protein 3, a chemokine that attracts macrophages; collagen type I alpha 1 chain; colony stimulating factor 3, controlling granulocyte function; C-X-C motif chemokine ligand 3, attracting neutrophils; coagulation factor XIII A chain; a subunit of coagulation factor XIII, playing a role in blood coagulation cascade; fibroblast growth factor 2 and 10, possessing mitogenic and cell survival activities; IL-6, playing a role in inflammation; integrin subunit alpha 5, subunit beta 3 and subunit beta 6, involved in cell surface adhesion and signaling; plasminogen activator that promote formation of plasmin; serpin family E member 1, inhibitor of tissue plasminogen activator; signal transducer and activator of transcription 3, influencing many genes taking part in growth and apoptosis; TIMP metallopeptidase inhibitor 1, an MMP inhibitor; cellular communication network factor 4, a signaling protein implicated in the Wnt/β-catenin inducible signaling pathway; and cellular communication network factor 2, a mitogen involved in cell adhesion [85].

Additionally, ROS also regulate the process of new blood vessel formation near the wound site [9]. Collagen formation during the tissue repair process is associated with pH in HDFs: pH > 7.50 downregulates collagen type I alpha 1 chain and upregulates MMP-1 by ROS production and MAPK signaling, whereas pH < 6.04 has less effects [86].

Excessive ROS production leads to impaired dermal fibroblast function, degenerative ECM proteins and weakened wound healing [9]. It was shown that ROS promote ATM serine/threonine kinase-mediated p53 signaling, increase dermal fibroblast apoptosis, and delay the process of cutaneous wound healing in mice under isocitrate dehydrogenase (NADP(+)) 2 (IDH2) deficiency, an important enzyme related with mitochondrial redox balance [87]. Another study indicates that improper regulation of ROS is followed by decreased SOD1 expression, which in turn provides for aged fibroblast dysfunction and wound healing impairment [88].

ROS production is elevated in diabetic wounds. Excessive glucose levels disturb intracellular redox homeostasis, weaken the antioxidant barrier, and lead to redox dysregulation [89]. In addition, defects in Nrf2 signaling during diabetes may disrupt the functions of fibroblasts [90]. Nrf2 regulates the expression of antioxidant genes including CAT, NADPH dehydrogenase quinone 1, glutathione reductase, and glutathione S-transferase in fibroblast cells [91]. Fibroblasts that lose their Nrf2 phenotype during wound healing are characterized by no changes in wound closure rate. However, those in which Nrf2 becomes activated are characterized by accelerated wound closure as well as senescence [67].

Additionally, excessive glucose levels predispose to the formation of advanced glycation end products (AGEs). Those products occur as a result of a non-enzymatic reaction between reducing sugar with protein amino groups. AGEs interact with their cell surface receptors leading to ROS production. The glycosylated matrix is associated with in vitro dermal fibroblasts, cell cycle arrest and apoptosis, as well as degenerative collagen [92]. Impaired wound healing under diabetes may be an effect of IGF1 resistance upon oxidative stress. Those effects are observed both in vitro and in vivo and are related to depletion of PI3K-Akt signaling and improper glucose disposal, respectively [93]. Fibroblasts obtained from human diabetic wounds exposed to AGEs are characterized by an excessive apoptosis rate; AGEs promote ROS production, followed by NLR family pyrin domain containing 3 (NLRP3) inflammasome induction [94].

Table 2 presents several plant extracts particularly phenolics and their in vitro effect on dermal fibroblast cells and migration capacity. Those extracts, apart from ROS scavenging properties, also exhibit tissue repair properties.

The migration rates of HDFs, T3T, or L929 cells after *Alternanthera sessilis*, *Lysimachia nummu-laria, Thymus sipyleus*, and *Alchemilla vulgaris* extracts treatment were assessed by the in vitro scratch wound healing assay. The cells were treatment with extract. Before incubation, after reaching appropriate cells confluence, a wound (“scratch”) was created in cell monolayer, in the middle of each well. Within the cell-free gap, the cell migration was visualized by microscopy. A percentage of the closed area was measured after incubation period and compared with the value obtained at 0 h. An increase in the percentage of the closed area indicated the migration of cells. Those extracts exhibit a remarkable migratory rate in fibroblast cell lines [77,95,97,98]. Mitogenic cell proliferation rate in HDFs exposed to *Cenostigma pluviosum* extract was evaluated by the BrdU incorporation assay [96]. The data indicate that higher cell proliferation and migration rate improve tissue regeneration [99].

One of the abovementioned extracts from *Alchemilla vulgaris* was tested in vivo in a human model of sodium lauryl sulfate (SLS) irradiated skin. The products were applied twice a day for seven days. Highest extent of wound closure was observed in treated skin [98].

Effective synthesis of ECM collagen by dermal fibroblasts is linked to proliferation and migratory rate and important to wound repair. Those effects are gained by treatment of cells with single phenolic compounds derived from plants. The viability of NIH-3T3 albino mouse fibroblastic cell line was improved by luteoin, a flavonoid isolated from *Tragopogon graminifolius*. That effect was related with increased cell population [100]. Myricetin-3-*O*-β-rhamnoside, a flavonoid obtained from *Parrotia persica*, promotes the migration of HDFs [101]. A higher fibroblast proliferation rate translates into increased collagen production, as was observed in HDFs treated with geraniin and furosin, an ellagitannin from *Phyllanthus muellerianus* [102]. Stimulation of collagen production was also observed in HDFs exposed to batatasin III, a stilbene obtained from *Dendrobium loddigesii* [103]. Another phenolic compound, 6-dehydrogingerdione, a phenolic alkenone isolated from the rhizomes of *Zingiber officinale*, induces both proliferation and migration of HDFs. Additionally, HDF treatment activates collagen production, inhibition of MMP-1 protein expression, and upregulation of TIMP-1 secretion [104]. These findings show that phenolic compounds synergistically accelerate fibroblasts and are effective in the wound healing process.

## 7. Modulation of ROS Levels in Fibroblasts by Phenolic Compounds and Their Role in the Aging Process

Skin aging is influenced by intrinsic and extrinsic factors. The former is connected with a physiological process that results in fine wrinkles, dry and thin skin, or gradual dermal atrophy. The latter is related to environmental factors like solar ultraviolet (UV) exposure, air pollution, poor nutrition and smoking, and results in coarse wrinkles, laxity, loss of elasticity, and a rough-textured appearance [105]. External aging is largely due to ROS from UV radiation [106]. ROS overproduction impairs collagen biosynthesis and activates collagenase [54], as well as elastin deposition, which is characteristic of photoaged skin [58]. HDFs exposed to increased level of endogenous ROS demonstrate reduced cell motility and deletions in mitochondrial DNA [107]. Mitochondrial dysfunction is closely linked to skin aging and a senescence phenotype [108].

Excessive ROS in the biological environment can lead also to lipid peroxidation (LPO), an important marker of photodamage. Hydroxyl radicals react with polyunsaturated fatty acids in the cell membrane, produce peroxyl radicals, and activate a chain reaction generating other reactive species, such as lipid hydroperoxides, which lead to alterations in membrane permeability and UV-related skin pathology [109]. Among all wavelengths, solar UVA radiation most effectively triggers the peroxidation response in cultured HDFs [110]. The level of HDF protection against LPO induced by UVA depends primarily on glutathione level [111].

Senescent cells are more common in aged human skin [112]. That state may be induced by ROS and lead to the activation of proinflammatory pathways like MAPKs and NF-κB. Senescent fibroblasts secrete a high amount of MMPs and less collagen [113]. The enhanced MMP activity disrupts the interaction between fibroblasts and ECM, resulting in a vicious circle effect and additional collagen proteolysis [74]. Studies show that irradiated senescent human dermal fibroblasts show downregulation of collagen genes, including collagen type VIII alpha 2 chain and collagen type V alpha 3 chain, and the upregulation of MMP genes and TLR pathway genes like TLR4. TLR4 stimulates ERK signaling, followed by mitochondrial dysfunction and increased levels of MMP-1 and IL-8 [114]. Another factor regulated by MAPK kinases are NF-κB/p65. In photoaged fibroblasts, the expression of COX-2 and an inflammatory cytokine regulated by NF-κB/p65 is significantly higher than in non-irradiated cells [115].

Elevated levels of ROS may be additionally induced by permanent exposure to the TNF-α overexpressed in irradiated skin, resulting in premature senescence of HDFs and inflammatory phenotype as well as a transient phosphorylation of p38 MAPK. ROS accumulation may be limited by antioxidative enzymes. Underexpression of genes taking part in the antioxidative response, such as heme oxygenase-1 (HO-1) and NAD(P)H:quinone oxidoreductase 1 (NQO1) regulated by Nrf-2, has been observed in fibroblasts exposed to UV [116]. The skin of mice with SOD gene knockdown in fibroblasts demonstrate accelerated aging, limited collagen production, dermal thickness, and accumulation of senescence phenotype [117].

ROS levels increase in human dermal fibroblasts following aging; a process related to the activation of the phosphatidylinositol-3-OH kinase (PI3K)/Akt pathway or reduced phosphatase and tensin homolog (PTEN) level. PTEN is an inhibitor of PI3K/Akt, which dephosphorylates phosphatidylinositol 3,4,5-triphosphate (PIP3), a molecule that is converted to phosphatidylinositol (3,4)-bisphosphate (PIP2) by PI3K and leading to Akt translocation [118]. Akt induction activates numerous downstream genes [119]. Another study, under diabetic conditions, also indicated greater ROS production and activation of PI3K/Akt pathway in dermal fibroblasts treated with basic fibroblast growth factor (bFGF) [120].

Table 3 presents several plant extracts with the main components belonging to the phenolics group and their capacity as anti-photoaging agents in fibroblast cell lines.

To evaluate the photoprotective effects of plant extracts, the fibroblasts were exposed to UVA or UVB light. UVA rays (320–400 nm) are capable of penetrating into the dermis where fibroblasts reside and to induce cell damage via generation of ROS, through the photoexcitation of endogenous photosensitizers [142]. UVB radiation exposure (290–320 nm) can also induce the production of ROS in dermal fibroblasts, including the hydroxyl free radical, superoxide anion, singlet oxygen and hydrogen peroxide [143], as well as reduced migration [144]. Then, cells were treated with extracts and incubated.

After irradiation of skin fibroblasts, the activity of catalase and superoxide dismutase decreases [145]. Some plant extracts derived from *Pterocarpus santalinus* [125], *Arachis hypogaea* [127], *Abelmoschus esculentus* [133], and *Syzygium aromaticum* [134] demonstrate stimulation of antioxidant defense by Nrf2 overexpression in fibroblast cell lines. *Byrsonima crassifolia* extract [132] prevented the decrease in reduced GSH levels in fibroblasts. ROS initiate chain reaction of lipid peroxidation in the cell membranes that is inhibited by *Nectandra cuspidate* [130] and *Nectandra hihua* [131] extracts. Moreover, ROS disturb the signal transduction pathways that are involved in the expression of genes, which regulate collagen metabolism. Due to signal transduction cascades, an overexpression of matrix metalloproteinases MMPs occurs. These enzymes catalyze the degradation of the corresponding proteins and their expression in fibroblasts are limited by *Toxicodendron vernicifluum* [121], *Gynura procumbens* [123], *Lithospermum erythrorhizon* [124], *Pterocarpus santalinus* [125], *Cassia fistula* [126], *Flemingia macrophylla* [128], *Hypericum olympicum* [129], *Epilobium angustifolium* [135], *Passiflora tarminiana* [136], *Rosa multiflora* [138], and *Crataegus pinnatifida* [139] extracts. Moreover, *Coffea arabica* extract limited inflammatory response in fibroblast cells by decrease COX-2 level and inhibition the translocation of NF-κB to the nucleus [140]. Without protection, skin layers are particularly susceptible to the sun damage. The DNA is often damaged by UV irradiation and can directly absorb UVB light. This facilitates the dimerization of pyrimidine bases, which can cause mutations and errors in DNA replication. Furthermore, UVA can also inhibit DNA repair. This effect and the described activation of MMPs and lipid oxidation increase the probability of premature skin ageing. DNA damages are reduced by Fragaria × ananassa extract in fibroblast cells [137]. In summary, plant extracts rich in polyphenols can play a photoprotective role and prevent excessive ROS production.

Some of the abovementioned extracts were tested in parallel in in vivo human/animal models. Application of *Nectandra cuspidata* extract into the skin of hairless mice (HRS/J) prior exposed to UV demonstrated inhibition of ROS production, reduction of MMP-2 and MMP-9 activity, and increased antioxidant defense by decreasing reduced glutathione (GSH) and CAT depletion. Additionally, the analysis of the skin surface showed limited appearance of sunburn cells, epidermal thickness, and skin edema [130]. *Coffea arabica* extract analysis restored the collagen content, inhibited NF-κB, IL-6 and MMP-1 expression, and reduced epidermal hyperplasia in Balb/c hairless mice [136]. The skin of SKH-1 hairless mice exposed to UV irradiation followed by Rosa multiflora extracts exhibit reduced epidermal thickening. Additionally, the levels of TNF-α, IL-6, and MMP-13 were reduced [140]. *Crataegus pinnatifida* limited epidermal thickening and dermal damage in a Balb/c mouse model after prior UV irradiation; treatment also limited ROS, NF-κB, MAPK, and MMP expression, and stimulated the production of type I procollagen [139]. Decrease in reduced GSH levels, IL-1β, and IL-6 secretion, and inhibition of MMP-9 were observed in UV-radiated hairless mice HRS/J exposed to *Byrsonima crassifolia* extract [132]. Swiss albino mice, UV-irradiated and treated with *Ilex paraguariensis* extract, demonstrated the inhibition of MMP-2 expression [122]. *Epilobium angustifolium* protect against erythema formation in UV-irradiated skin in human participants [135].

Isolated compounds also play a pivotal role in fibroblast function stimulation. Pedunculagin, ellagitannin isolated from *Quercus mongolica*, increased type I procollagen synthesis and inhibited its effect on MMP-1 in UV-irradiated HDFs [138]. The same effects were observed following (−)-catechin treatment obtained from *Ulmus davidiana* var. japonica; it also limited ROS accumulation and COX-2 expression and decreased the production of other proinflammatory cytokines like IL-1β and IL-6 [146]. Another two anthocyanins (cyanidin 3-galactoside and cyanidin 3-lathyroside) from *Acanthopanax divaricatus* var. albeofructus increase antioxidant enzyme activities (SOD and CAT) in UV-irradiated HDFs [147]. Finally, incubation with 2,3,5(2,4,5)-tricaffeoylaltraric acid and 2,4(3,5)-dicaffeoylglucaric acid isolated from *Galinsoga parviflora* reduces ROS production, increases GSH level and Nrf2 overexpression, and induces heme oxygenase 1 (HO-1) in UV-irradiated HDFs [148]. All those compounds are good candidates to ameliorate skin aging.

## 8. Conclusions

Plant extracts rich in phenolic compounds and isolated chemicals have great potential for ROS scavenging and skin care. The review collects literature on the effects of phenolic compounds on fibroblasts in in vitro studies over the last 10 years. Phenolics stimulate collagen production in fibroblasts. In addition, phenolics limit the expression of MMPs via downregulation of the AMPK pathway and suppression of NF-κB translocation to the nucleus followed by decreased level of COX-2 and anti-inflammatory effects under oxidative stress conditions. Finally, phenolics enhance cell antioxidant defense and enzyme activities via Nrf2 overexpression. Topical application of selected extracts prevent dermal damage. These results suggested that phenolic compounds can be useful for supporting fibroblast function, accelerating wound healing, and protecting against UV-induced photoaging. However, research on fibroblasts is currently lacking and more studies are needed.

## Figures and Tables

**Table 1:** Selected plant extracts from different species with identified phenolic compounds and their in vitro effect on dermal fibroblast cells and ROS production.

Name of the Families	Name of the Species (Common Names)	Part of the Plant	Type of Extract (Concentrations)	Identified Bioactive	Cell Lines	Mechanisms of Action	Ref.
Annonaceae	*Annona muricata* L. (soursop)	leaves	aqueous (12.5 to 200 µg/mL)	quercetin 3-glucoside, rutin, chlorogenic acid, catechin, and gallic acid	L929 fibroblasts exposed to 750 µmol/L H_2_O_2_	Reduced ROS production	[76]
Onagraceae	*Fuchsia magellanica* Lam. (hardy fuchsia)	leaves	aqueous/ethanolic (1000 µg/mL)	various phenolic acid, flavonoid, and anthocyanin derivatives	3T3 fibroblasts exposed to 1 mM 2,2′-azobis(2-amidinopropane) dihydrochloride (AAPH)	Reduced ROS production	[77]
Poaceae	*Oryza sativa* L. (rice)	panicles	ethyl acetate (0.1 to 100 µg/mL)	gallic, protocatechuic, chlorogenic, caffeic, syringic, p-coumaric, ferulic, sinapic and rosmarinic acids, vanillin, and quercetin	HDFs exposed to 150 µmol/L H2O2	Reduced oxidative stress	[78]
Rosaceae	*Pourthiaea villosa* (Thunb.) Decne. (oriental photinia)	leaves	ethanolic (25 to 100 µg/mL)	p-coumaric acid, caffeic acid, chlorogenic acid, patuletin, catechin, epicatechin, eriodictyol, naringenin, quercetin, and quercetin derivatives	HDFs exposed to 1 mmol/L H2O2	Reduced ROS production	[79]
Salicaceae	*Populus nigra* L. (Lombardy poplar)	whole plant	aqueous (25 to 200 μg/mL)	caffeic and p-coumaric acids	HDFs exposed to 100 µmol/L AAPH	Reduced ROS production	[80]

**Table 2:** Selected plant extracts from different species with identified phenolic compounds and their in vitro effect on dermal fibroblast cells and migration capacity.

Name of the Families	Name of the Species (Common Names)	Part of the Plant	Type of Extract (Concentrations)	Identified Bioactive	Cell Lines	Mechanisms of Action	Ref.
Amaranthaceae	*Alternanthera sessilis* (L.) R.Br. ex DC. (sessile joyweed)	stems	Ethanolic (12.5 to 50 μg/mL)	2,4-dihydroxy-2,5-dimethyl-3(2H)-furan-3-one, hexadecanoic acid, 2-1,2,4-trioxolane,3-phenyl, palmitate ethyl, and L-glutamic acid	HDFs and diabetic HDFs	Increased migratory rate	[95]
Fabaceae	*Cenostigma pluviosum* (DC.) Gagnon & G.P.Lewis	stem bark	ethanolic	pyrogallol, gallic acid, gallic acid methyl ester, ellagic acid, corilagin, 1,4,6-tri-*O*-galloyl-glucose, tellimagrandin I, 1,2,3,6-tetra-*O*-galloyl-glucose, mallotinic acid, tellimagrandin II, 1,2,3,4,6-penta-*O*-galloyl-glucose, geraniin, and mallotusinic acid	HDFs	Increased cell proliferation rate	[96]
Lamiaceae	*Thymus sipyleus* Boiss.	aerial parts	ethanolic (50 to 200 μg/mL)	luteolin-7-*O*-glucoside	3T3 fibroblasts	Increased migratory rate	[97]
Primulaceae	*Lysimachia nummularia* L. (creeping jenny)	leaves	ethanolic (10 to 50 μg/mL)	various phenolic acid, flavonoid, and anthocya-nin derivatives	3T3 fibroblasts	Increased migratory rate	[77]
Rosaceae	*Alchemilla vulgaris* L.	whole plant	ethanolic	kaempferol, luteolin, apigenin-7-Oglucoside, luteolin-7-*O*-glucoside, isoquercetin, and ellagic acid	L929 fibroblasts	Increased migratory rate	[98]

**Table 3:** Selected plant extracts from different species with identified phenolic compounds and their in vitro effect on dermal fibroblast cells as anti-photoaging agents.

Name of the Families	Name of the Species (Common Names)	Part of the Plant	Type of Extract (Concentration)	Identified Bioactive	Cell Lines	Mechanisms of Action	Ref.
Anacardiaceae	*Toxicodendron vernicifluum* (Stokes) F.A.Barkley (Chinese lacquer)	rhus	methanolic (1 to 50 μg/mL)	gallic acid, 2-(ethoxymethoxy)-3-hydroxyphenol, fustin, a fustin isomer, tetragalloyl glucose, pentagalloyl glucose, fisetin, sulfuretin, a sulfuretin isomer, and butein	HDFs exposed to UVA radiation	Reduced MMP-1 expression	[121]
Aquifoliaceae	*Ilex paraguariensis* A.St.-Hil. (mate)	leaves	ethanolic (40 to 400 μg/mL)	chlorogenic acid and caffeic acid	HFF-1 fibroblasts exposed to UVB radiation	Not cytotoxic for non-irradiated cells; photostable and non-phototoxic for radiated cells	[122]
Asteraceae	*Gynura procumbens* Merr.	leaves	ethanolic (1 to 20 μg/mL)	quercetin 3-*O*-rutinoside and isobioquercetin, kaempferol 3-*O*-rutinoside	HDFs exposed to UVB radiation	Reduced MMP-1 and MMP-9 production	[123]
Boraginaceae	*Lithospermum erythrorhizon* Siebold & Zucc. (Lithospermum)	whole plant	methanolic (0.1 to 10 mg/mL)	rabdosiin, rosmarinic acid, lithospermic acid, lithospermic acid B, salvianolic acid A, and acetylshikonin, isomers of lithospermic acid, shikonofuran E, b-hydroxyisovalerylshikonin, isobutylshikonin, b,b-dimethylacrylshikonin, and isovalerylshikonin	HDFs exposed to UVA radiation	Reduced MMP-1 expression	[124]
Fabaceae	*Pterocarpus santalinus* L.f. (red sandalwood)	heartwoods	ethanolic (10 μg/mL)	taxifolin, quercetin, and naringenin	HDFs exposed to UVB radiation	Reduced MMP-1, MMP-3, IL-6, AP-1 and MAPKs expression Increased Nrf2 activity	[125]
Fabaceae	*Cassia fistula* L. (golden shower)	flowers	butanolic (25 to 200 μg/mL)	vanillic acid and protocatechuic acid, gallic acid, coumaric acid, ferulic acid, and chlorogenic acid, catechin	HDFs	Increased collagen and hyaluronic acid synthesis Reduced ollagenase and MMP-2 activity	[126]
Fabaceae	*Arachis hypogaea* L. (peanut)	sprout	ethanolic (0.005% to 2.5% from the stock)	trans-resveratrol	HDFs exposed to UVB radiation	Reduced ROS production Increased Nrf2 activity	[127]
Fabaceae	*Flemingia macrophylla* (Willd.) Kuntze ex Merr.	stems	aqueous (10 to 500 μg/mL)	daidzin, genistin,	Hs68 fibroblasts exposed to UVB radiation	Reduced elastase and collagenase activity, MAPKs, MMP-1, MMP-3, MMP-9 expression Increased type I procollagen expression	[128]
Hypericaceae	*Hypericum olympicum* L.	flowering aerial parts	methanolic (0.5 to 1.5 mg/mL)	chlorogenic acid and quercetin glycosides (rutin, hyperoside, isoquercitrin)	HDFs exposed to UVB radiation	Reduced MMP-9 concentrations	[129]
Lauraceae	*Nectandra cuspidata* Nees & Mart.	leaves	ethyl acetate fraction (6.54 μg/mL)	epicatechin, isovitexin, and vitexin	L-929 fibroblasts exposed to UVB radiation	Reduced ROS production, LPO inhibition	[130]
Lauraceae	*Nectandra hihua* (Ruiz & Pav.) Rohwer (shinglewood)	leaves	ethyl acetate fraction (10 μg/mL)	quercitrin, avicularin, juglalin, afzelin, and astragalin	L929 fibroblasts exposed to UVB radiation	Reduced ROS production, LPO inhibition	[131]
Malpighiaceae	*Byrsonima crassifolia* (L.) Kunth (maricao cimun)	leaves	ethanolic—partially purified (0.6 to 5 µg/mL)	catechin, epigallocatechin gallate, quercetin 3-O-β-D-glucopyranoside	L929 fibroblasts exposed to UVB radiation	Prevented the decrease in reduced GSH levels	[132]
Malvaceae	*Abelmoschus esculentus* (L.) Moench (okra)	fruits	ethyl acetate fraction (5 to 30 µg/mL)	rutin	HDFs exposed to UVB radiation	Prevented: UV-induced depletion of endogenous enzymatic antioxidants Reduced oxidative DNA damage, ROS production, apoptotic changes Increased Nrf2 activity	[133]
Myrtaceae	*Syzygium aromaticum* (L.) Merr. & L.M.Perry (clove)	clove buds (5 to 40 µg/mL)	methanolic	flavonoid-enriched fraction: quercetin, kaempferol, gallic acid	HDFs exposed to UVB radiation	Prevented: UV-induced depletion of endogenous enzymatic antioxidants Reduced xidative DNA damage, ROS production, apoptotic changes Increased Nrf2 activity	[134]
Onagraceae	*Epilobium angustifolium* L.	aerial parts	Isopropylalcohol (10 μg/mL)	gallic acid, oenothein B, chlorogenic acid, myricetin-3-*O*-hexoside, myricetin-3-*O*-pentoside, myricetin-3-*O*-rhamnoside, quercetin-7-*O*-glucuronide, quercetin-3-Opentoside, kaempferol-3-*O*-hexoside, kaempferol-7-O-glucuronide; and kaempferol-3-*O*-rhamnoside	UV-irradiated HDFs	Reduced MMP-1, hyaluronidase 2 gene expression Increased TIMP-1, TIMP-2 gene expression	[135]
Passifloraceae	*Passiflora tarminiana* Coppens & V.E. Barney (banana passionflower)	fruits	aqueous (2.5 to 10 μg/mL)	dimeric proanthocyanidins of flavan-3-ols, flavone derivatives	HDFs exposed to UVB radiation	Reduced ROS production, MMP-1 expression Increased procollagen production	[136]
Rosaceae	*Fragaria × ananassa* (Duchesne ex Weston) Duchesne ex Rozier (strawberry)	fruits	Methanolic (0.05 to 0.5 mg/mL)	anthocyanins	HDFs exposed to UVA radiation	Reduced DNA damages	[137]
Rosaceae	*Rosa multiflora* Thunb. (multiflora rose)	flowers	ethanolic (1 to 10 μg/mL)	quercitrin and hyperin	HDFs exposed to UVB radiation	Reduced MMP-1 expression Increased type I procollagen expression	[138]
Rosaceae	*Crataegus pinnatifida* Bunge (Chinese haw)	fruits	Ethanolic (5 to 10 μg/mL)	chlorogenic acid, procyanidin B2, and epicatechin	HDFs exposed to UVB radiation	Reduced MMP-1 expression, ROS production	[139]
Rubiaceae	*Coffea arabica* L. (Arabian coffee)	leaves	Methanolic (1 to 50 μg/mL)	chlorogenic acid	Hs68 cells exposed to UVB radiation	Reduced ROS production, COX-2 level, translocation of NF-κB to the nucleus	[140]
Rubiaceae	*Ixora parviflora* Lam.	leaves	methanolic (1 to 50 μg/mL)	chlorogenic acid	Hs68 cells exposed to UVB	Reduced ROS production	[141]

## Abbreviations

ACTA2	actin alpha 2
AGEs	advanced glycation end products
Bfgf	basic fibroblast growth factor
CAT	catalase
CCL	C-C motif chemokine ligand
CX3CL	C-X3-C motif chemokine ligand
CXCL	C-X-C motif chemokine ligand
CXCR	C-X-C motif chemokine receptor
DLK1	delta-like noncanonical Notch ligand 1
DPP4	dipeptidyl peptidase-4
ECM	extracellular matrix
EMT	epithelial-mesenchymal transition
G-CSF	granulocyte colony-stimulating factor
GM-CSF	granulocyte/macrophage colony-stimulating factor
GPx	glutathione peroxidase
HDFs	human dermal fibroblasts
HO-1	heme oxygenase-1
IDH2	isocitrate dehydrogenase (NADP(+))
IGF1	insulin like growth factor 1
IKK	inhibitory kappa kinase
IL	interleukin
INF γ	interferon
ITGA1	integrin subunit alpha 1
Keap1	Kelch-like ECH associated protein 1
LPO	lipid peroxidation
MAF	musculoaponeurotic fibrosarcoma
MAPKs	mitogen-activated protein kinases
MET	mesenchymal-epithelial transition
MMPs	matrix metalloproteinases
NF-kB	nuclear factor kappa-light-chain-enhancer of activated B cells
NLRP3	NLR family pyrin domain containing 3
NQO1	NAD(P)H:quinone oxidoreductase 1
Nrf2	nuclear factors (erythroid-derived 2), factor 2
PDGFR-α	platelet derived growth factor receptor alpha
PI3K	phosphatidylinositol-3-OH kinase
PTEN	reduced phosphatase and tensin homologs
SCA1	spinocerebellar ataxia type 1
SOD	superoxide dismutase
TGF-β	transforming growth factor
TIMPs	tissue inhibitors of metalloproteinase
TLR	toll-like receptors
TNF-α	tumor necrosis factor
